# Bridging health literacy and physical literacy for sustainable health

**DOI:** 10.3389/fpubh.2025.1699666

**Published:** 2025-11-05

**Authors:** Camilla Eriksson, Johannes Hedlund, Maria Harder, Lena Almqvist, Lisa Borglund

**Affiliations:** ^1^Department of Public Health Sciences, Mälardalen University, Västerås, Sweden; ^2^Sustainable Regional Development, Region Sörmland, Nyköping, Sweden; ^3^Department of Caring Science, Mälardalen University, Västerås, Sweden; ^4^Department of Psychology, Mälardalen University, Västerås, Sweden

**Keywords:** health literacy, physical literacy, sustainability, health, health interventions, public health

## Abstract

In this paper, we propose that physical literacy should be conceptualized in relation to, and as interconnected with, health literacy rather than as a distinct and separate form of literacy, as is often presented. Furthermore, we contend that this approach should be more centrally integrated into health interventions that target health in general, as well as specific aspects aimed at determinants of health, such as physical activity. Rather than focusing on isolated actions or behaviours, a literacy-centred model empowers individuals with the knowledge, skills, and confidence needed for sustained engagement in healthy practices. Integrating these literacies within intervention models bridges the gap between knowing and doing. This offers a holistic pathway to promoting lifelong relationships and responsibility for health and improving population health outcomes. We also want to draw attention to the importance of aiming to increase literacy at higher levels, as it is a variable that influences the relationship between health and poor social and economic conditions. However, it is important to engage in the difference between using literacies to displace responsibility on the individual and engaging in literacies as a component in interventions as an approach to sustainable action.

## Health as a holistic concept

The concept of health is inherently holistic and multidimensional, with definitions that vary across disciplines, contexts, and cultures (1, 2). Nevertheless, the majority of definitions view health as a holistic concept, emphasizing the importance of human flourishing, achieving significant life goals, and living a good life (3–6). The most prevalent definition of health is that proposed by the World Health Organization (WHO), which defines it as “a state of complete physical, mental, and social well-being and not merely the absence of disease or infirmity” (6). This definition is widely used but also intensely debated, as the phrase “complete physical, mental, and social well-being” is seen as an utopian, ableist, and a static state of health. Health can be understood as a positive construct and resource for human potential and human sustainability. This understanding encompasses the notion of human flourishing, which includes experiencing happiness, pursuing and achieving meaningful goals (4, 7), growing and developing as a person, and having important and meaningful social relationships with others (8, 9). Ultimately, this holistic and positive perspective frames health as a dynamic, lifelong process that fosters resilience, personal growth, and the capacity to thrive sustainably.

The concept of health, and thus human flourishing, encompasses all lifestyle habits and the social determinants of health (10). Despite the holistic definition and understanding of health, which posits that all aspects of health are interconnected and that the body and mind are inextricably linked, health interventions frequently focus on a single determinant, such as physical activity, without considering the literacy of that specific health determinant. Consequently, despite the promising efficacy of physical activity interventions at the individual and group levels, there has been no discernible increase in physical activity at the population level (11, 12). A plausible explanation is that interventions only embed skills to obtain health information and apply to a specific activity in a specific situation, but one does not develop skills to *engage* and *transfer* actions or knowledge to different settings and situations (1). Additionally, guidelines on healthy lifestyles provide important health-related information but fail to inspire action. To achieve human sustainability and flourish, we must shift the focus to one that redefines health interventions to empower individuals by focusing on developing specific health-related literacies. In practice, this entails concentrating on elements such as knowledge, understanding, motivation, and confidence in one’s competencies. By equipping individuals and populations with the ability to obtain, evaluate, and utilize health-related information independently, which is commonly referred to as health literacy (13), a healthy lifestyle becomes sustainable and personally meaningful.

## Defining and understanding health literacy

Health literacy is defined as “access, understand, appraise and use information and services in ways that promote and maintain good health and well-being” (13). This implies the attainment of a level of knowledge, personal skills, and confidence to take action to improve personal and community health through changes in personal lifestyles and living conditions. Nutbeam and Lloyd (1, p.162) further put health literacy as a personal skill in relation to specific contexts and situations, *“personal skills that are mediated by the environment in which these skills are to be applied. […] Recognizing the impact of situational demands and complexities also focuses attention on simplifying communication and reducing the complexities of the health systems that people must navigate. Both represent important methods for addressing the challenges posed by poor health literacy in the health system and in the wider community”*.

Nutbeam and Lloyd (1) describe three levels of health literacy: functional health literacy, interactive health literacy, and critical health literacy. Functional health literacy is a basic-level, task-oriented skill that is sufficient to obtain health information and apply it to specific, prescribed activities (such as taking medication). Interactive health literacy enables individuals not only to extract information and derive meaning from different forms of communication and apply that information in different and changing circumstances but also to extend the information by interacting with others and making decisions. Critical health literacy is the most advanced level that can be applied to critically analyse information from a variety of sources and in relation to a greater range of health determinants. These skills allow greater control over situations and events that can impact health. The contemporary conceptualization of health literacy encompasses disease prevention and health promotion at both the individual and population levels, as well as situational and personal determinants. From a health promotion perspective on health literacy, Sørensen et al. (14) define the highest level of health literacy as “the ability to make informed decisions about health determinants in the social and physical environment.” In this context, informed decision-making represents the ultimate outcome of health literacy.

Additionally, there is a social gradient in health literacy, which decreases with increased financial deprivation, lower education, and age. This gradient reflects significant disparities in access to health knowledge and resources among different socioeconomic and demographic groups, with lower levels of health literacy often found in disadvantaged populations. Importantly, research has also revealed gender-based differences in health literacy, with men having slightly lower health literacy than women do (1, 2). Thus, people may experience varying levels of access, understanding, and utilization of health information due to differing social roles, health-seeking behaviours, and exposure to health education. These disparities limit individuals’ capacity to make informed and empowered decisions about their health. In this way, health literacy extends beyond personal skills and represents a vital social determinant of health that influences how equitably health resources and benefits are distributed across society.

Despite the existence of numerous national and international guidelines pertaining to the social determinants of health and individual-level lifestyles, including physical activity (15–17), individuals frequently fail to act on these recommendations. Adhering to recommendations such as these requires a higher level of health literacy and thus fails to inspire those who need it the most, namely, individuals with low levels of health literacy (often individuals in vulnerable social and economic positions). All health determinants can be subsumed under the umbrella of health literacy, including physical activity. However, while knowledge and motivation are essential components of health literacy, they lack components for specific health determinants and are not sufficient to engage individuals in physical activity. To effectively influence population-level activity patterns, it is necessary to consider physical literacy, which is a more detailed form of literacy that emphasizes the valuation and relationship with physical activity as a component of health literacy. This is where physical literacy adds complexity, understanding, and specific factors that facilitate the transformation of knowledge into sustained action.

## Defining and understanding physical literacy

The idea of physical literacy varies across different countries. For example, Physical and Health Education Canada (18) defines physical literacy with a focus on skills and the ability to “move with competence in a wide variety of physical activities that benefit the development of the whole person.” Australia has a more holistic approach, including both learning and applying movements, defining it as “lifelong holistic learning acquired and applied in movement and physical activity contexts” (19). England takes a broader view, describing physical literacy as “our relationship with movement and physical activity throughout life” (20). Together, these definitions reflect differing emphases—on competence, holistic development, or the relational and social meaning of movement—illustrating the conceptual diversity and contextual adaptation of physical literacy. Further on, we will refer to physical literacy as defined by Whitehead (21), which posits that it comprises “motivation, confidence, physical competence, knowledge and understanding to value and take responsibility for engagement in physical activities throughout one’s lifetime” (21). In this perspective, physical literacy comprises four interrelated elements—physical (physical competence), cognitive (knowledge and understanding), affective (motivation and confidence), and behavioural (active engagement in movement for life)—that promote the *relation* to physical activity, that is, meaning-making and commitment to pursue physical activity throughout life. This further implies that an individual may not necessarily be physically active consistently yet possess the requisite competence and motivation to resume an active lifestyle following an inactive period. Moreover, an individual with physical literacy is capable of identifying alternative forms of physical activity. For example, a fit person who has sustained an injury is unable to resume regular exercise and becomes physically inactive is considered to have low physical literacy. In contrast, someone who values and has a meaningful relationship with movement, and therefore is able to engage in other forms of physical activity, is regarded as having high physical literacy. Hence, physical literacy can serve as a protective factor against a decline in physical activity, particularly in the context of external events (such as the COVID-19 pandemic) that impede regular physical activity habits (22). This is likely because individuals with higher levels of physical literacy can adapt and find alternative movement contexts and ways to maintain their physical activity and do so because of their *valuation* and *relation* to physical activity.

## Unpacking the intersections and divergences of health and physical literacy

There are notable differences between health literacy and physical literacy. First, health literacy is broad and encompasses health as a whole and relates primarily to healthcare, disease prevention, and health promotion, whereas physical literacy focuses on a specific health determinant. While health literacy focuses primarily on levels of knowledge and *knowing*, and first, at the higher level of health literacy, putting knowledge into action, physical literacy focuses on internal factors such as motivation and confidence, which relate more closely and directly to *doing.* This is evident in the aspects of physical literacy that relate to valuation and relation to physical activity, which are lacking in health literacy. Hence, physical literacy corresponds to a minimum level of interactive health literacy, as it, by definition, encompasses *relation* and *meaning-making.* Given that health literacy is a broad literacy that includes navigating a health system and appraising and using information about all lifestyle habits, it does not provide sufficient competence to pursue physical activity. Physical literacy, on the other hand, includes these exact aspects and is thus a mediator between health literacy and physical activity. By further elucidating these connections, we can facilitate and advocate for health promotion and bridge the knowing-doing gap through a comprehensive understanding of the competence, motivation, and confidence of physical activity.

Meta-analyses of physical activity interventions in primary care show that the physical activity that health professionals advocate and prescribe has insufficient evidence, inconsistent effects, and unsustainable behaviour (23–25). Interventions aiming to increase physical activity often focus on *physical activity behaviour* as an outcome at a functional health literacy level but fail to target interactional and critical health literacy, as well as physical literacy, as a foundation for maintaining physical activity over time.

The physical literacy perspective includes social, and enjoyment factors related to physical activity, encouraging individuals to engage in and sustain such behaviour. Unfortunately, health professionals who encounter patients needing to modify their lifestyle habits often apply a health literacy approach. In contrast, a physical literacy approach has primarily been used in the development of pedagogy in sports and physical exercise (26, 27), which may not effectively reach the most physically inactive populations. Linking multiple settings increases the likelihood of decreasing persistent physical inactivity in a population (28) and increases the capacity to combat physical inactivity at the societal level. Moreover, there is a tendency to focus on behaviour change, placing all responsibility on the individual rather than the setting or context, which also explains the lack of maintenance of physical activity behaviour interventions.

Physical literacy is concerned with a specific aspect of health literacy but encompasses the intrinsic factors that influence an individual’s capacity to engage in a range of movement contexts in a meaningful manner. When viewed from the perspective of its role as an antecedent to physical activity, physical literacy is purely a health promotion concept. Consequently, health literacy involves knowledge about what and why physical activity should be performed, whereas physical literacy represents the transition from knowledge to action. This highlights an important opportunity to incorporate essential training in physical literacy for health professionals. Such training could enhance the effectiveness of physical activity prescriptions and support the development of sustainable physical activity behaviours. While critical health literacy has potential in this context—particularly in fostering informed decision-making—it may fall short in addressing the motivational, emotional, and social dimensions of movement that physical literacy emphasizes.

**Figure 1:**
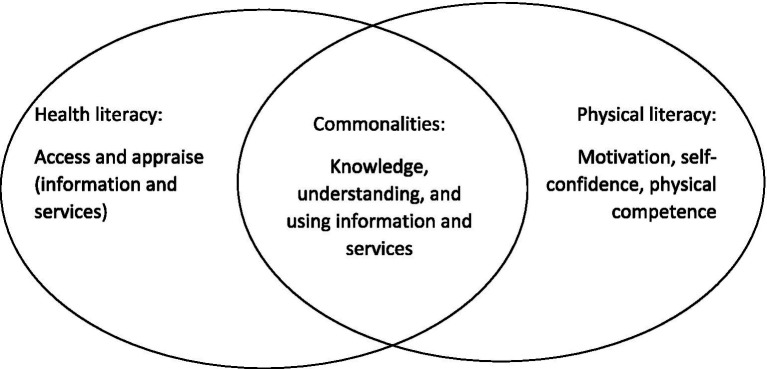
Illustrating how the conceptual overlap and differences based on applied definitions.

Nevertheless, both health literacy and physical literacy have a holistic approach to health and aim to empower individuals and influence their actions through both individual skills and supportive environments for health. Both physical literacy and health literacy share common ground, as they involve elements of knowledge, learning, informed decision-making, and the promotion of an active lifestyle. However, important differences between them also exist, and these distinctions influence how actions are shaped depending on which literacy framework is applied. Physical literacy, for instance, places greater emphasis on the motivational, emotional, and social dimensions of movement, which are often overlooked in health literacy approaches. Figure 1 illustrates the similarities and differences between health literacy and physical literacy definitions.

Health literacy encompasses those aspects that, in physical literacy, are regarded as “knowledge and understanding.” Health literacy can be seen as primarily promoting *knowing,* and physical literacy primarily promotes *doing.* However, in addition to health literacy, physical literacy adds important aspects influencing actions, e.g., self-confidence and physical competence. To date, health literacy is widely used in public health and health care, while physical literacy is mostly limited to different sports contexts. Nevertheless, we argue that health literacy and physical literacy must be seen as interconnected instead of widely separated. Integrating health literacy and physical literacy within the same scientific discipline has the potential to significantly enhance societal impact—not only through school education (beyond the scope of physical education), but also within healthcare, public health interventions, and community planning.

By adopting a physical literacy-centered approach, interventions can move beyond isolated behaviours and instead empower individuals with the knowledge, skills, and confidence needed for sustained engagement in healthy practices. For example, sedentary patients may be more likely to adhere to physical activity prescriptions when emotional and social dimensions of movement—such as enjoyment and meaning-making—are emphasized, rather than focusing solely on health outcomes. We further argue for a similar shift in physical education and school settings, where fostering enjoyment and meaningful engagement in movement should complement the teaching of health-related knowledge.

At the societal level, incorporating physical literacy into the operationalization of physical activity guidelines and interventions can help reframe the focus from individual preferences to broader contextual and environmental factors. While individual tailoring remains important—especially in clinical settings—population-level strategies should prioritize inclusive, socially and emotionally engaging environments that support lifelong physical activity.

## Conclusion

We propose that physical literacy should be regarded as a whole subset of health literacy. These factors are important to connect, both theoretically and in interventions. The competence to make informed decisions about health is broad and nonspecific, given the multitude of health determinants it encompasses. In healthcare and public health, it can enhance adherence to physical activity prescriptions and support population-level strategies that address environmental and social determinants of health. Crucially, this approach shifts the focus from placing responsibility solely on individuals to designing systems and interventions that empower communities. As such, integrating these literacies is not just a theoretical alignment—it is a practical necessity for advancing equitable and effective health promotion.

Most importantly, we argue that physical literacy is an important key to creating supportive environments that enable changing physical inactivity behaviour at the group and population levels and should be considered simultaneously and complementary to health literacy. Bridging health literacy and physical literacy thus opens up opportunities for several disciplines and refocuses actions on increased physical activity at a population level. However, we encourage further discussions on this topic.
